# Levels of Hope, Stigma, Psychological Vulnerability, and Positive Mental Health: A Descriptive Study of Eighth- and Ninth-Grade Adolescents

**DOI:** 10.3390/healthcare13111257

**Published:** 2025-05-26

**Authors:** Maria José Carvalho Nogueira, Delfina Teixeira

**Affiliations:** 1 School of Health of the Polytechnic Institute of Santarém at Santarém, 2001-904 Santarém, Portugal; 2 Comprehensive Health Research Centre (CHRC), Évora University, 7004-516 Évora, Portugal; 3 RISE-Health, Porto University, 4099-002 Porto, Portugal; delfinateixeira@hotmail.com; 4 Shcool of Health of Trás-os-Montes e Alto Douro University, 5000-801 Vila Real, Portugal

**Keywords:** hope, stigma, positive mental health, nursing, adolescents, literacy

## Abstract

**Background/Objectives**: This study aimed to characterize adolescents’ levels of hope, stigma, psychological vulnerability, and positive mental health in a school context. **Methods:** A cross-sectional descriptive study was conducted in a non-probabilistic sample of 189 adolescents from eighth–ninth grade in 2021. During the citizenship discipline, adolescents filled out an online self-completion questionnaire for data collection, containing all measurement instruments: Hope Thermometer, Attribution Questionnaire, Psychological Vulnerability Scale, and Positive Mental Health Questionnaire. **Results:** The majority were men (55.1%) with a mean age of 14 years. Overall, adolescents have acceptable levels of hope (M = 8; SD = 2.58), a high level of stigma (M = 25.6; SD = 5.23), satisfactory positive mental health (M = 118.3; SD = 14.8), and moderate psychological vulnerability (M = 15.2; SD = 6.4). **Conclusions:** Findings support educational practices and policies that target personalized intervention to promote and improve hope and positive mental health in adolescents. These data are relevant to getting ahead and designing more positive mental health behavior programs to reinforce adolescents’ modifiable healthy aspects and positive mindsets.

## 1. Introduction

Adolescence is widely recognized as a critical and vulnerable period for mental health due to the complex physical and mental health changes that occur in adolescents during this developmental transition period [1,2,3,4]. Mental health problems in children and adolescents have increased in recent years, with one in five children showing evidence of these issues. Research shows that mental health problems are common among adolescents, often associated with shame and bullying [5]. Furthermore, high levels of mental health problems persistence are one of the main predictors of mental problems in adulthood [3,6]. Adolescents’ well-being, accomplishments, and educational success are related to hope, positive mental health, and psychological vulnerability [7,8]. Feeling hopeful is an important condition for adolescents’ health, well-being, educational success, and attainment [7]. Adolescents need to be positive and feel hopeful to achieve their developmental goals [9,10]. Also, the literature shows that there is a strong association between self-esteem, positive mental health (PMH) [11], and hope [12]. Yet, limited research has examined the relation of these variables in adolescence. Hope focuses on goal attainment cognition, whereas behavioral hope focuses on actions required for goal attainment [9]. Besides this abstract construct, hope has been studied across various disciplines to describe, explain, and predict the association between hope and human functioning, and this is seemingly vital. Bryce found that cognitive hope significantly predicted achievement, school engagement, anxiousness, and stress in adolescents [9]. Also, evidence has been established of positive and negative relations between hope and adolescents’ mental health and emotional, psychological, and social well-being [10,13]. A recent study shows that hope remains similar across grades six to ten, but a decrease occurs before the transition to high school, and school performance stress may contribute to this decrease [7]. Also, hope is positively associated with optimal mental health outcomes like life satisfaction and negatively associated with psychological distress and, as a cognitive-motivational strength, it is among the useful internal resources that foster positive adaptation and promote positive mental health in adolescents [9,10].

Furthermore, adolescents’ positive mental health (PMH) during adolescence and youth is particularly important for healthy development, and evidence establishes a relationship between positive mental health literacy (MHL) and mental well-being among adolescents [2,3]. In recent years, experts have shown an increasing concern, not only due to the significant prevalence of mental health disorders in this population but also due to the early-age onset of the first episode of these disorders—before 14 years of age [14].

Additionally, mental health disorders and their recognition are not specifically included in the school curriculum, and youth have low levels of knowledge and awareness of mental health literacy [1,2,3,4]. The lack of MHL increases adolescents’ perceived mental health stigma, which in turn is the major barrier to finding help and getting professional treatment [5,9,15,16]. Recent studies show that increasing education and awareness, being compassionate and understanding to those experiencing mental health problems, and linking students to persons were important strategies to reduce stigma-related attitudes [12,17,18]. Thus, MHL programs in the school context are scarce but fundamental for the healthy development of children and young people [19,20].

In Portugal, there are a few studies characterizing the levels of positive mental health and psychological vulnerability (PV) in adolescents up to sixteen years of age, but we failed to find any study that characterizes the levels of hope and stigma in this age group. Data regarding levels of hope, stigma, psychological vulnerability, and PMH in adolescents and their relations are important to enlighten researchers and practitioners about their specificities, but they are scarce. Gathering this information can offer an opportunity to design accurate interventions to improve hope, reduce stigma, and promote PMH among adolescents through a multidisciplinary approach to guarantee youth mental health protection and positive perspectives [12]. Therefore, we start with the research question: What are the levels of hope, stigma, psychological vulnerability, and positive mental health among eighth and ninth-grade adolescents?

Thus, this study aims to describe hope, stigma, psychological vulnerability levels and positive mental health in eighth–ninth-grade adolescents in a school context.

## 2. Materials and Methods

### 2.1. Study Design

This study is comprised in the scope of the quantitative research paradigm and is a cross-sectional, descriptive design study performed in a non-probabilistic sampling method. The STROBE statement checklist [21] was used as a guide for writing this article.

### 2.2. Setting

An online self-completion questionnaire (Google^®^ Forms) was used, containing all the variables under study. The form has been configured in compliance with the General Data Protection Regulation, with the option to not collect respondents’ emails or ask for identifiable data enabled. Parents and teachers were informed about the existence of support offices and the school’s Psychology and Guidance Services (SPO), and at the end of the form, an email and contact telephone number were provided where they could request confidential help. Data collection took place during the regular teaching period in schools, from January to March 2021, in public schools in the North Region of Portugal, during the citizenship discipline, as previously agreed upon with the teacher. All participants completed the questionnaire in the presence of the principal investigator (PI). During data collection, the adolescents’ doubts about the instruments were addressed by the PI of the study.

### 2.3. Participants

Participants were selected through a convenience sample of adolescents enrolled in eleven eighth and ninth-grade classes in two public schools, all of whom voluntarily agreed to participate in the study. From a total population of approximately 1200 students, adolescents were deemed eligible if they met the following criteria: (a) their parents or legal guardians had provided written informed consent and (b) the students themselves agreed to participate voluntarily. Exclusion criteria included adolescents with cognitive impairment, those without parental or guardian consent, and those who declined to participate. Measuring instruments were not administered to students who did not meet these inclusion criteria. A total of 191 online questionnaires were completed, of which 2 were excluded due to duplication. Consequently, the final sample comprised 189 participants with fully completed responses.

The study was conducted in accordance with the ethical principles outlined in the Declaration of Helsinki and the Oviedo Convention, and received approval from the Ethics Committee and the Board of Directors of the participating institutions (Ref. UI&D 01/2021-ESECVP-AT). Additionally, permission was obtained from the authors of the instruments used. To ensure ethical data collection, an email was sent to the school board directors containing a link to the informed consent form (hosted on Google^®^ Forms), which was then forwarded to the parents or legal guardians. All procedures were conducted in full compliance with the General Data Protection Regulation (GDPR). Participants and tutors were previously informed by email about the purpose and implications of the study, possible risks/benefits, ethical aspects, and their right to withdraw at any time by not submitting the form, and assured about the anonymization of the data. All the adolescents and the parents or tutors gave written informed consent to participate and use the data for research purposes. Participants were guaranteed the anonymity of the data collected.

### 2.4. Data Sources/Measurement

To respond to the research question outlined, adolescents were evaluated in a single moment. Concerning demographic characteristics, we used four characterization variables (sex, age, class, and nationality) and thirteen behavioral variables (mental illness, relationships, exercise, sleep, diet, medication, and substance consumption). To assess hope, stigma, psychological vulnerability, and positive mental health levels, we used the measurement instruments:

The Hope Thermometer [12] is used to measure the level of perception of hope. Hope Thermometer is a 1-to-10-point Likert-type scale, where 1 represents a total absence of hope and 10 indicates the greatest level of hope ever experienced. Higher scores reflect a higher perceived level of hope.

The Attribution Questionnaire (AQ-8-C) adolescent version [22,23] to assess stigma stereotypes about mental illness. The AQ-8-C have 8 stereotype items rated on a 9-point Likert-type scale (1 = no or not at all to 9 = a lot or completely). The result produces a representative score for each of the stereotypes, with stigma being directly proportional to the score value. Results greater than 1 imply the existence of stigma. In the present study, the AQ-8-*C*’s Cronbach’s alpha was satisfactory.

The Psychological Vulnerability Scale (PVS) Portuguese version [24] was used to assess psychological vulnerability, which is an inadequate cognitive pattern (perfectionism, dependence, need for external sources of approval, widespread negative attributions). PVS is a six-item self-administered one-dimensional structure instrument that rates on a 5-point Likert scale from 1 = does not describe me at all to 5 = describes me very well. Total scores range from 6 to 30, with higher scores indicating greater psychological vulnerability and values above 15 indicating psychological vulnerability. Portuguese version internal consistency was adequate (*Cronbach alpha* = 0.73), and 5-week stability was excellent (Test–retest, r = 0.88, *p* < 0.0001) [24]. Adolescents took an average of nineteen minutes to complete the questionnaire.

The Positive Mental Health Questionnaire (PMHQ) [25] was used to assess participants’ positive mental health. This self-administered instrument comprises 39 items rated on a 4-point Likert scale (1 = “Always or almost always” to 4 = “Rarely or never”). Of these, 20 items are positively worded, and 19 are negatively worded. The PMHQ is structured into six factors: F1—Personal Satisfaction; F2—Prosocial Attitude; F3—Self-Control; F4—Autonomy; F5—Problem-Solving and Personal Achievement; and F6—Interpersonal Relationship Skills. The total PMHQ score is calculated by summing all item responses, yielding a range from 39 to 156 points, with higher scores indicating better levels of positive mental health. The Portuguese version of the PMHQ demonstrates excellent internal consistency, with a total Cronbach’s alpha of 0.92 and subscale alphas ranging from 0.60 to 0.84. Test–retest reliability over a two-month interval showed strong stability (r = 0.98) [25]. For qualitative interpretation, PMHQ scores are categorized into three levels–Languishing (39–78), Intermediate (79–117), and Flourishing (118–156)—with higher scores reflecting more favorable mental health status [26].

### 2.5. Data Analysis

Statistical data analyses were conducted using IBM SPSS Statistics Version 27 (IBM Corp., Armonk, NY, USA) for Windows. A threshold of 10% missing data was established as the exclusion criterion for questionnaires. Descriptive and exploratory statistical techniques were applied, including absolute and relative frequencies, means, and standard deviations, to describe the variables based on their typology (qualitative/quantitative). Measures of central tendency (mean and mode) were used to identify typical values in the dataset, while measures of dispersion (minimum and maximum values, variance, and standard deviation) assessed the variability of the data [27,28]. A preliminary test of variable distribution (Shapiro–Wilk test) indicated that the sample did not follow a normal distribution (*p* < 0.001). Accordingly, non-parametric tests were used in inferential analyses to evaluate associations between nominal variables and the instruments applied. Internal consistency was assessed using Cronbach’s alpha coefficients based on standardized items. Cronbach’s alpha values range from 0 to 1, with higher values indicating greater reliability. Statistical significance was set at *p* < 0.05 [28].

## 3. Results

### 3.1. Participant’s Characteristics

The sample consisted of 189 adolescents, mostly male (44.9%), with an average age of 13.97 years (SD = 2.5), ranging from a minimum age of 12 years to a maximum of 18 years, attending eighth–ninth grade (Table 1). The majority were Portuguese (97%) and attended the ninth grade (56.4%). Regarding health behavior, the majority reported positive health behavior and had no prior psychological or psychiatric follow-up (69.6%), nor relatives with mental illnesses (88.4%). Adolescents reported that they sleep a sufficient number of hours per day (M = 7.99; SD = 6.5), the majority reported practicing physical exercise and eating fruits and vegetables every day in their diet, but they reported eating a low average of meals per day (M = 3.96; SD = 1.3; Mode = 4). The majority reported engaging in recreational activities (62.3%), and 17.4% had an affective relationship with pets. As expected, the most significant affective relationships mentioned by participants were friends, followed by family members. Less than 5% admitted to smoking, and less than 10% admitted to alcohol consumption. Table 1 summarizes the participants’ characteristics and details.

Results from the descriptive analysis of the measures Hope Thermometer, Attribution Questionnaire (Stigma), Positive Mental Health Questionnaire levels, and Psychological Vulnerability Scale in the sample are summarized in Table 2.

### 3.2. Hope Thermometer Levels

Adolescents perceived their hope levels as satisfactory (M = 8; SD = 2.58), with the majority scoring above the possible midpoint for the Hope Thermometer.

### 3.3. Attribution Questionnaire AQ-8-C—Stigma

Results also show a high score at *AQ-8-C* (M = 25.6; SD = 5.23), since all items scored up to one, indicating high levels of stigma in the sample. The lowest levels of *AQ-8-C* were obtained at Segregation, Avoidance, and Shame items. AQ-8-C’s Cronbach’s alpha in the sample was acceptable, although low (Table 2).

### 3.4. Psychological Vulnerability Scale

The results obtained from the PVS show moderate psychological vulnerability (M = 15.2; SD = 6.4) in the sample. PVS’s Cronbach’s alpha in the sample was good. Details are presented in Table 2.

### 3.5. Positive Mental Health Questionnaire Levels

Concerning the PMH, the results from *PMHQ* display that adolescents report good levels of PMH (M= 118.3; SD = 14.8), indicating a state of flourishing in the sample. The highest levels were recorded at sub-scales F1: Personal Satisfaction and F5: Problem-Solving. The sub-scales F3: Self-Control and F4: Autonomy obtained the lower levels. As shown in Table 2, participants’ overall levels of PMH were F lourishing (scores 118 to 156), followed by Intermediate (scores 79 to 117), and four at the Languishing level (scores 39 to 78). PMH’s Cronbach’s alpha in the sample was excellent (Table 2).

## 4. Discussion

This study aimed to describe hope, stigma, positive mental health, and psychological vulnerability levels in a sample of eighth-ninth-grade adolescents. Overall, participants report perceptions of positive behaviors regarding sleep, physical exercise, and diet (eating fruit and vegetables every day), and this result must be interpreted as a positive health behavior, as reported in previous studies [3,29,30]. Also, participants reported very low levels of tobacco and alcohol consumption, a tendency in line with a recent study that shows that 90.8% of participants stated that they do not consume alcoholic drinks or smoke (96.5%). This behavior is a good and important sign, as healthier behaviors are significant positive correlates in adolescents’ better mental health outcomes [11,31].

As expected, the most significant affective relationships mentioned by adolescents were friends, followed by family members [32]. Affective relationships increase self-esteem and strengthen the experience of social inclusion, which has a stronger association with adolescents’ mental well-being [11]. Furthermore, friendships with peers can provide a unique developmental context in which adolescents learn how to manage conflicts, negotiate, develop empathic concern, as well as develop skills. The evidence highlights the important contributions of friendship, strong peer relationships, and family support in preventing adolescents’ mental health problems [11,33]. For adolescents, the main role of friendship is predominantly an important psychological schema that satisfies important social needs, companionship and intimacy [34]. Schemas are mental frameworks (efficient or inefficient mechanisms) that help individuals organize and interpret experiences and information about the world [13]. The findings reinforce the importance of disseminating this knowledge within school contexts to foster a positive and safe environment that supports the development and strengthening of interpersonal relationships during this critical stage of adolescence [13]. Also, data can now be used and support future studies to find hypothetical relations between adolescent cultural characteristics and variables studied, particularly hope and stigma.

The present study contributes to the understanding of hope, a variable that has been little studied in adolescents, despite its great relevance for better understanding this population and designing more suitable interventions. In Portugal, our results added novel data, underlining that adolescents’ levels of hope are satisfactory and highlighted that this is good news. In fact, during school transitions, students experience disturbances in various domains of development (cognitive and socio-emotional), often leading to difficulties in adaptation [10], with hopeful future expectations playing an important role that helped them to transition positively through the period of adolescence [35]. Finding good levels of hope in the sample is important once the evidence shows that hope is a protective factor against depressive and anxiety symptoms in adolescents [7]. Hope is also associated with better mental well-being in transversal and longitudinal design studies [36]. Adolescents’ hope may bolster their abilities to negotiate the demands associated with systematic change and therefore ameliorate some of the difficulties associated with educational transitions [36]. Understanding students’ levels of hope can assist educators in identifying optimal moments to reinforce goal-setting behaviors, as hope is considered a teachable and malleable construct. Therefore, our findings may now support future studies in establishing a positive relationship between hope and PMH, since most participants achieved overall positive levels of PMH. Enhancing hope may support students during the critical transition from middle to high school, a period that presents both challenges and opportunities for positive adjustment [7]. Specifically, adapting school environments during these transitions through a multidisciplinary approach can foster hope, shape expectations, and promote positive outlooks in the face of mental health challenges [7,9]. An example of school-based mental health strategies may include the following: regular screening of students’ hope levels using simple tools like the Hope Thermometer to identify those in need of support; the integration of hope-building interventions into the curriculum (e.g., goal-setting workshops, resilience training, or social-emotional learning activities); teacher and staff training to recognize early signs of hopelessness or distress and respond with appropriate referral or support; and collaboration among educators, psychologists, and families to create consistent and positive messages about coping, future orientation, and emotional well-being. Embedding these elements into a whole-school approach, institutions can create more supportive, inclusive, and mentally healthy environments, particularly during sensitive developmental transitions. Our results can be interpreted as positive support for the reinforcement of hope in the school context; however, future studies must investigate, in larger samples, the relations between hope, positive mental health, and adolescent adaptation through the school path to add robustness to these results.

Results show high levels of stigma in the sample, which is quite disturbing, as stigma discourages individuals from seeking and obtaining both informal and professional help [18,24,37]. Adolescents’ stigma towards mental health problems is a stronger predictor of help-seeking intentions, and this behavior is a vital matter because adolescents highly value what friends and peers think about their behavior [17]. Adolescents with limited or inaccurate mental health information (low literacy) held more stigmatizing attitudes about individuals with mental health disorders [5,9,16]. Although the current study contributes valuable insights, the low Cronbach’s alpha obtained for the *AQ-8-C* scale suggests that findings should be interpreted with caution. Further validation studies with larger and more diverse samples are needed to ensure the reliability and applicability of this instrument in adolescent populations. Evidence shows that a comprehensive psychoeducational mental health promotion intervention in a school context improves the students’ ability to recognize mental health problems and reduce stigma [5]. This evidence systematically emphasizes the importance of deepening the knowledge about stigma in broader and longitudinal studies, for instance, to understand how adolescents deal with stigma, shame, and positive mental health [18,37].

In the sample, Segregation, Avoidance, and Shame parameters were the stigma parameters with the lowest levels. We consider this data important for designing more personalized interventions for young people in the future, aimed at increasing their knowledge, understanding, and open-mindedness regarding mental health prejudices, especially younger adolescents have higher stigma rates and lower levels of mental health literacy (MHL) [3]. Some authors recommend that MHL promotion interventions are needed in schools for young people with and without mental disorders, that they must include younger students and be focused on increasing their ability to understand and engage in their mental health issues [5,20]. Additionally, interventions aimed at promoting young people’s good mental health have the potential to increase knowledge and skills for maintaining a healthy lifestyle in adulthood [5,20] once the level of mental health literacy increases with age [3]. In Portugal, limited research has been conducted on stigma among adolescents, despite the importance of this knowledge for designing and implementing educational programs aimed at stigma reduction. Thus, our results are aligned with the need to implement programs based on five aspects: “mental health literacy training”, “integration and coordination of stakeholder organizations”, “resources and facilities”, “continuous assessment”, and “provision of information” [24].

The results obtained from the PVS show a moderate psychological vulnerability (PV), with girls reporting higher levels of PV than boys. These findings are in line with recent ones, showing that PV increases with age, with late adolescents reaching high levels of PV [6,33]. Psychological vulnerability is an idiosyncratic structural characteristic of individuals [38], and higher PV is associated with a lower level of knowledge about the factors that promote good mental health [3]. Thus, because adolescents are a vulnerable group, our findings add novel data to support more accurate and tailored interventions focused on this particular age to promote adolescents’ mental health literacy. Innovative approaches are a key responsibility of the health services, schools, as well as the entire society [39], so our results can contribute to and reinforce the progressive worldwide focus on adolescent mental health promotion.

The positive mental health (PMH) levels of the sample are good. The majority scored at Flourishing overall levels of PMH, and four adolescents scored at Languishing levels, in line with previous studies in similar populations [38,40]. This positive result may be related to healthy positive health behaviors (sleep, physical exercise, and diet) reported by participants, despite COVID-19 confinement [3]. Recent studies have shown that higher levels of positive mental health are significantly associated with greater well-being, higher self-esteem, stronger character traits, increased hope and kindness, and enhanced social inclusion [2,3,11]. Onnela and colleagues [5] reported that implementing mental health promotion interventions among young people can effectively enhance their knowledge and skills for maintaining a healthy lifestyle throughout adolescence and adulthood. Post-intervention, students demonstrated improved recognition of mental health disorder symptoms. Notably, the intervention particularly increased boys’ ability to identify conduct disorders and was perceived as beneficial, fostering greater knowledge, understanding, and openness regarding mental health. Given these findings, the mental health of children and young people must remain a priority for policymakers and health professionals. Strengthening strategies for promoting PMH and combating stigma is essential for fostering healthier, more resilient communities [5].

Renwick defends that adolescents can identify how they pursue PMH and well-being by developing age- and culture-appropriate community mental health strategies. Therefore, it is important to highlight that training adolescents on how to obtain and maintain good mental health is an asset and should be an integral component of adolescents’ school education [41].

Mental health promotion interventions aim to increase the ability of individuals to understand mental health issues and engage in self-care. Interventions must reinforce adolescents’ modifiable healthy aspects and positive mindsets through workshops about mental health issues (sleep hygiene, stress, aggressiveness, well-being, and relaxation strategies) to strengthen resiliency, mental health literacy, and decrease barriers to help-seeking behavior. Also, inclusive assistance, counselling, pedagogical support, and sports programs should be promoted. Our results can contribute to increasing awareness of the imperative need to promote the mental health of adolescents through a comprehensive approach involving the whole of society. Psychiatric nurses can collaborate with school nurses to provide school-based educational interventions to increase adolescent mental health resilience, reduce stigma and increase help-seeking behaviors [33].

Finding innovative approaches to improving mental health among adolescents in a school context is a key responsibility of the health services, schools, as well as the entire society [39]. Therefore, our results can contribute to reinforcing the progressive worldwide focus on adolescent mental health promotion. In Portugal, given the existing data gaps concerning stigma and hope within this age group, the information provided by this study is both important and groundbreaking. As such, the current research contributes to expanding existing knowledge in this field, offering valuable insights that can support and inform future studies.

Some limitations that may constrain the interpretation of the results must be addressed. Firstly, probabilistic sampling and the sample size limit the representativeness and the generalizability of the findings. Secondly, the social desirability bias is introduced by the effect of the self-completion instruments used. Therefore, future studies should employ a larger probabilistic sample and incorporate regression analysis or structural equation modelling to explore predictive relationships between hope, stigma, and mental health outcomes among adolescents.

## 5. Conclusions

The research showed that adolescents report perceptions of positive behaviors regarding sleep, physical exercise, and diet, and mention friends and family as the most significant affective relationships. They also report satisfactory levels of hope, high levels of stigma, a good level of positive mental health, and moderate psychological vulnerability.

These data are particularly relevant to getting ahead and designing more positive mental health behavior programs that can be used to develop tailored intervention strategies and psychological support to maintain or increase adolescents’ positive mental health, prevent stigmatizing attitudes, and minimize emotional suffering.

Findings support educational practices and policies that target student hope, stigma, and positive mental health before the high school transition to potentially buffer student stress and promote high school achievement. Further studies are needed to validate and strengthen these findings, with a particular focus on deepening the understanding of the relationships between hope, stigma, and mental health, ideally using larger samples.

## Figures and Tables

**Table 1:** Demographic characteristics of the sample (N = 189).

Variables		N	%
Sex	Men	47	55.1
	Female	42	44.9
Age	M = 13.97; SD = 2.5		
Class	8th grade	40	43.5
	9th grade	49	56.5
Nationality	EUA	1	1.4
	Brazil	1	1.4
	Canada	1	1.4
	Portugal	85	94.2
	Senegal	1	1.4
Have you ever had any psychological or psychiatric follow-up?	No	58	69.6
	Yes	31	30.4
Do you have relatives with mental illnesses?	No	81	88.4
	Yes	8	11.6
Hours of sleep per day	M = 7.99; SD = 6.5		
Do you sleep enough for your needs?	No	10	14.5
	Yes	79	85.5
Do you take any sleeping medication?	No	89	100.0
	Yes	0	0
Do you take medication regularly for any mental health issues?	No	88	98.6
	Yes	1	1.4
Do you exercise regularly?	2 times per week	35	46.2
	3 times per week	31	40.4
	No practice	24	23.4
Do you consider your diet healthy?	No	6	8.7
	Yes	83	91.3
Do you eat fruits and vegetables daily?	No	6	8.7
	Yes	83	91.3
Do you have any recreational activities?	No	36	37.7
	Yes	53	62.3
Do you have an affective relationship?	Pet	12	17.4
	Family	33	34.8
	Friends	40	43.5
	Boy/girlfriend	4	4.3
Are you satisfied with your affective relationship?	No	4	5.8
	Yes	85	94.2
Do you smoke?	No	86	97.1
	Yes	3	2.9
Do you consume alcoholic beverages?	No	83	92.8
	Yes	6	7.2

**Table 2:** Descriptive analysis of Hope Thermometer, AQ-8-C, Total PMHQ and sub-scales and PVS in the sample n= 189.

Hope Thermometer	Min	Max	Mean	SD
With 1 feeling of total hopelessness and 10 being the most hopeful you have ever felt, how do you feel today?	1	10	8.00	2.58
**AQ-8-C items**				
1. Would you feel sorry for John?	1	9	6.41	2.35
2. Would you think John is dangerous?	1	9	1.96	2.23
3. How afraid would you be of John?	1	9	1.74	1.82
4. I think João is to blame for his mental illness.	1	9	1.43	1.46
5. I think John should be in a special class for children with problems, not a normal one like mine.	1	9	3.09	1.51
6. How angry would you feel with John?	1	9	1.58	2.61
7. How likely are you to help John with his schoolwork?	1	9	7.09	1.48
8. Would I try to stay away from John after school?	1	9	2.32	2.33
**Total AQ-8-C α = 0.497**	8	72	25.6	5.23
**PVS items**				
1. If I do not achieve my goals, I feel like a failure as a person	1	5	2.55	1.13
2. I feel entitled to better treatment from others than I generally receive	1	5	2.52	1.218
3. I am frequently aware of feeling inferior to other people	1	5	2.23	1.41
4. I need approval from others to feel good about myself	1	5	2.12	1.40
5. I tend to set goals too high and then become frustrated trying to reach them	1	5	2.80	1.26
6. I often feel resentful when others take advantage of me	1	5	3.00	1.49
**Total PVS α = 0.797**	6	30	15.2	6.4
**PMHQ Sub-scales**				
F1: Personal satisfaction	11.00	32.00	26.1	5.2
F2: Prosocial attitude	13.00	20.00	17.5	2.0
F3: Self-control	8.00	20.00	15.0	3.1
F4: Autonomy	6.00	20.00	15.0	3.3
F5: Problem-solving	20.00	36.00	28.8	4.1
F6: Interpersonal relations	13.00	26.00	19.9	2.2
**Total PMHQ α = 0.911**	86.00	147.00	118.3	14.8
**PMHQ Global Levels:**				
High level (*flourishing*) 144 (76.2%) Intermediate level 41 (21.7%) Low level (*languishing*) 4 (2.1%)				

*α* = *Cronbach’s alpha*; AQ-8-C—Attribution Questionnaire (adolescent version); PVS—Psychological Vulnerability Scale; PMHQ—Positive Mental Health Questionnaire.

## Data Availability

The original contributions presented in this study are included in the article. Further inquiries can be directed to the corresponding author.

## References

[1] Lam L.T. (2014). Mental health literacy and mental health status in adolescents: A population-based survey. Child Adolesc. Psychiatry Ment Health.

[2] Bjørnsen H.N., Espnes G.A., Eilertsen M.-E.B., Ringdal R., Moksnes U.K. (2019). The Relationship Between Positive Mental Health Literacy and Mental Well-Being Among Adolescents: Implications for School Health Services. J. Sch. Nurs..

[3] Nobre J., Calha A., Luis H., Oliveira A.P., Monteiro F., Ferré-Grau C., Sequeira C. (2022). Mental Health Literacy and Positive Mental Health in Adolescents: A Correlational Study. Res. Public Health.

[4] Pretorius C., Chambers D., Coyle D. (2019). Young people’s online help-seeking and mental health difficulties: Systematic narrative review. J. Med. Internet Res..

[5] Onnela A., Hurtig T., Ebeling H. (2021). A psychoeducational mental health promotion intervention in a comprehensive schools: Recognising problems and reducing stigma. Health Educ. J..

[6] Nogueira M.J., Sequeira C., Sampaio F. (2021). Gender differences in mental health, academic life satisfaction and psychological vulnerability in a sample of college freshmen: A cross-sectional study. J. Gend. Stud..

[7] Fraser A.M., Bryce C.I., Alexander B.L., Fabes R.A. (2021). Hope levels across adolescence and the transition to high school: Associations with school stress and achievement. J. Adolesc..

[8] Nogueira M.J., Sequeira C. (2020). Preditores de Bem-estar Psicológico em Estudantes do Ensino Superior. Supl. Digit. Rev. Rol. Enferm..

[9] Bryce C.I., Alexander B.L., Fraser A.M., Fabes R.A. (2020). Dimensions of hope in adolescence: Relations to academic functioning and well-being. Psychol. Sch..

[10] Jiang X., Otis K.L., Weber M., Huebner E.S., Gallagher M.W., Lopez S.J. (2018). Hope and adolescent mental health. The Oxford Handbook of Hope.

[11] Ahrnberg H., Appelqvist-Schmidlechner K., Mustonen P., Fröjd S., Aktan-Collan K. (2021). Determinants of Positive Mental Health in Adolescents–A Cross-Sectional Study on Relationships between Positive Mental Health, Self-Esteem, Character Strengths and Social Inclusion. Int. J. Ment. Health Promot..

[12] Querido A., Tomás C., Carvalho D. (2016). O estigma face à doença mental nos estudantes de saúde. Rev. Port. Enferm. Saúde Ment..

[13] Schmid K.L., Lopez S.J. (2011). Positive pathways to adulthood: The role of hope in adolescents’ constructions of their futures. Adv. Child Dev. Behav..

[14] Patalay P., Gage S.H. (2019). Changes in millennial adolescent mental health and health-related behaviours over 10 years: A population cohort comparison study. Int. J. Epidemiol..

[15] Sequeira C., Sampaio F. Enfermagem em Saúde Mental; Lidel Enfe: 2020. https://m.lidel.pt/pt/catalogo/ciencias-da-enfermagem/enfermagem/enfermagem-em-saude-mental/.

[16] Dias C., Valentim O., Seabra P., Nogueira M.J. (2020). Intervenções promotoras de esperança em enfermagem de saúde mental e psiquiátrica—uma scoping review. Rev. Port. Enferm. Saúde Ment..

[17] Nearchou F.A., Bird N., Costello A., Duggan S., Gilroy J., Long R., McHugh L., Hennessy E. (2018). Personal and perceived public mental-health stigma as predictors of help-seeking intentions in adolescents. J. Adolesc..

[18] Vidourek R.A., Burbage M. (2019). Positive mental health and mental health stigma: A qualitative study assessing student attitudes. Ment. Health Prev..

[19] International Council of Nurses (2019). Programa Nacional para a Saúde Mental.

[20] Fusar-Poli P., Salazar de Pablo G., De Micheli A., Nieman D.H., Correll C.U., Kessing L.V., Pfennig A., Bechdolf A., Borgwardt S., Arango C. (2020). What is good mental health? A scoping review. Eur. Neuropsychopharmacol..

[21] von Elm E., Altman D.G., Egger M., Pocock S.J., Gøtzsche P.C., Vandenbroucke J.P. (2014). The Strengthening the Reporting of Observational Studies in Epidemiology (STROBE) Statement: Guidelines for reporting observational studies. Int. J. Surg..

[22] Corrigan P. (2004). How stigma interferes with mental health care. Am. Psychol..

[23] Corrigan P., Markowitz F., Watson A., Rowan D., Kubiak M. (2003). An attribution model of public discrimination towards persons with mental illness. J. Health Soc. Behav..

[24] Nogueira M.J., Barros L., Sequeira C. (2017). Psychometric Properties of the Psychological Vulnerability Scale in Higher Education Students. J. Am. Psychiatr. Nurses Assoc..

[25] Sequeira C., Carvalho J.C., Sampaio F., Sá L., Lluch-Canut T., Roldán-Merino J. (2014). Avaliação das propriedades psicométricas do questionário de saúde mental positiva em estudantes portugueses do ensino superior. Rev. Port. Enferm. Saúde Ment..

[26] Kuettel A., Pedersen A.K., Larsen C.H. (2021). To Flourish or Languish, that is the question: Exploring the mental health profiles of Danish elite athletes. Psychol. Sport. Exerc..

[27] Ferrando P.J., Lorenzo-Seva U. (2018). Assessing the Quality and Appropriateness of Factor Solutions and Factor Score Estimates in Exploratory Item Factor Analysis. Educ. Psychol. Meas..

[28] Watkins M.W. (2018). Exploratory Factor Analysis: A Guide to Best Practice. J. Black Psychol..

[29] Alves S.P. (2018). Criação e Validação de um Programa Promotor de Saúde Mental Positiva em Adolescentes [Creation and Validation of a Program to Promote Positive Mental Health in Adolescents].

[30] Nogueira M.J., Dias C., Alves P., Teixeira D. (2023). Promoção da literacia e saúde mental positiva: Estudo quase-experimental com adolescentes em contexto escolar. Rev. ROL Enfermería..

[31] Garcia I.R., Sequeira C.A.d.C. (2016). Saúde Mental Positiva em Adolescentes.

[32] Van Droogenbroeck F., Spruyt B., Keppens G. (2018). Gender differences in mental health problems among adolescents and the role of social support: Results from the Belgian health interview surveys 2008 and 2013. BMC Psychiatry.

[33] Rodriguez L.M., Moreno J.E., Mesurado B. (2021). Friendship Relationships in Children and Adolescents: Positive Development and Prevention of Mental Health Problems. Psychiatry and Neuroscience Update.

[34] Zamirinejad S., Hojjat S.K., Moslem A., MoghaddamHosseini V., Akaberi A. (2018). Predicting the Risk of Opioid Use Disorder Based on Early Maladaptive Schemas. Am. J. Mens. Health.

[35] Padilla-Walker L.M., Millett M.A., Memmott-Elison M.K. (2020). Can helping others strengthen teens? Character strengths as mediators between prosocial behavior and adolescents’ internalizing symptoms. J. Adolesc..

[36] Rosa AGS Literacia em Saúde Mental em Adolescentes. Desenvolvimento de um Instrumento de Avaliação em Adolescentes. Universidade do Porto; 2018..

[37] Shahraki-Mohammadi A., Panahi S., Ashouri A., Sayarifard A. (2022). Important Systemic Factors for Improving Adolescent Mental Health Literacy. Iran. J. Psychiatry..

[38] Suryaputri I.Y., Mubasyiroh R., Arfines P.P., Rachmalina R., Idaiani S., Sitorus N., Rosha B.C., Khotimah E.N., Setiyawati D. (2023). The Effects of a School-Based Mental Health Program on Students’ Knowledge, Behavior, and Depression: A Quasi-Experimental Study in Four Indonesian High Schools. J. Popul. Soc. Stud..

[39] Sequeira C., Carvalho J.C., Gonçalves A., Nogueira M.J., Lluch-Canut T., Roldán-Merino J. (2019). Levels of Positive Mental Health in Portuguese and Spanish Nursing Students. J. Am. Psychiatr Nurses Assoc..

[40] Renwick L., Pedley R., Johnson I., Bell V., Lovell K., Bee P., Brooks H. (2021). Conceptualisations of positive mental health and wellbeing among children and adolescents in low- and middle-income countries: A systematic review and narrative synthesis. Health Expect..

[41] Özbıçakçı Ş., Salkim Ö.Ö. (2024). The predictors of mental health literacy among adolescents students. Arch. Psychiatr. Nurs..

